# Supplementary material for: HIVprotI: an integrated web based platform for prediction and design of HIV proteins inhibitors
Source: J Cheminform. 2018 Mar 9;10:12. doi: 10.1186/s13321-018-0266-y (PMC5845081; doi:10.1186/s13321-018-0266-y)
Supplement: Supplementary file 2 — Additional file 2. Source code of HIVProtI web server. [file 13321_2018_266_MOESM2_ESM.zip › HIVprotI_Source-code/hivprotI/weka-3-6-6/doc/weka/core/pmml/package-tree.html]

weka.core.pmml Class Hierarchy


---


|  |  |  |
| --- | --- | --- |
| **Overview** \| **Package** \| Class \| **Tree** \| **Deprecated** \| **Index** \| **Help** \| **Weka's home** | |  |
| **PREV**   **NEXT** | **FRAMES**    **NO FRAMES**     **All Classes** |  |


---


## Hierarchy For Package weka.core.pmml

**Package Hierarchies:**: All Packages

---

## Class Hierarchy

- java.lang.Object
  - weka.core.pmml.**Expression** (implements java.io.Serializable)
    - weka.core.pmml.**Constant**- weka.core.pmml.**Discretize**- weka.core.pmml.**FieldRef**- weka.core.pmml.**NormContinuous**- weka.core.pmml.**NormDiscrete**
    - weka.core.pmml.**FieldMetaInfo** (implements java.io.Serializable)
      - weka.core.pmml.**DerivedFieldMetaInfo** (implements java.io.Serializable)- weka.core.pmml.**MiningFieldMetaInfo** (implements java.io.Serializable)- weka.core.pmml.**TargetMetaInfo** (implements java.io.Serializable)
      - weka.core.pmml.**FieldMetaInfo.Interval** (implements java.io.Serializable)- weka.core.pmml.**FieldMetaInfo.Value** (implements java.io.Serializable)- weka.core.pmml.**Function** (implements java.io.Serializable)
            - weka.core.pmml.**BuiltInArithmetic**- weka.core.pmml.**BuiltInMath**- weka.core.pmml.**BuiltInString**- weka.core.pmml.**DefineFunction**
            - weka.core.pmml.**MappingInfo** (implements java.io.Serializable)- weka.core.pmml.**MiningSchema** (implements java.io.Serializable)- weka.core.pmml.**PMMLFactory**- weka.core.pmml.**PMMLUtils**

## Interface Hierarchy

- weka.core.pmml.**PMMLModel**

## Enum Hierarchy

- java.lang.Object
  - java.lang.Enum<E> (implements java.lang.Comparable<T>, java.io.Serializable)
    - weka.core.pmml.**FieldMetaInfo.Value.Property**- weka.core.pmml.**FieldMetaInfo.Interval.Closure**- weka.core.pmml.**FieldMetaInfo.Optype**

---


|  |  |  |
| --- | --- | --- |
| **Overview** \| **Package** \| Class \| **Tree** \| **Deprecated** \| **Index** \| **Help** \| **Weka's home** | |  |
| **PREV**   **NEXT** | **FRAMES**    **NO FRAMES**     **All Classes** |  |


---
